# mRNA and Protein Expression in Human Fetal Membrane Cells: Potential Biomarkers for Preterm Prelabor Rupture of the Fetal Membranes?

**DOI:** 10.3390/ijms242115826

**Published:** 2023-10-31

**Authors:** Emmeli Mikkelsen, Berthold Huppertz, Ripudaman Singh, Katarina Ravn, Lotte Hatt, Mogens Kruhøffer, Rheanna Urrabaz-Garza, Niels Uldbjerg, Ramkumar Menon, Torben Steiniche

**Affiliations:** 1 Department of Clinical Medicine, Aarhus University, Palle Juul-Jensens Blvd. 11, 8200 Aarhus, Denmark; emmeli.mikkelsen@clin.au.dk (E.M.); uldbjerg@dadlnet.dk (N.U.); 2 Department of Obstetrics and Gynaecology, Aarhus University Hospital, Palle Juul-Jensens Blvd. 99, 8200 Aarhus, Denmark; 3 Division of Cell Biology, Histology and Embryology, Gottfried Schatz Research Center, Medical University of Graz, Neue Stiftingtalstrasse 6, 8010 Graz, Austria; berthold.huppertz@medunigraz.at; 4 ARCEDI Biotech Aps, Tabletvej 1, 7100 Vejle, Denmark; rs@arcedi.com (R.S.); kr@arcedi.com (K.R.); loha@arcedi.com (L.H.); 5 BioXpedia, Palle Juul-Jensens Blvd. 82, 8200 Aarhus, Denmark; mkruhoffer@bioxpedia.com; 6 Division of Basic Science and Translational Research, Department of Obstetrics and Gynecology, University of Texas Medical Branch at Galveston, 301 University Blvd., Galveston, TX 77555, USAra2menon@utmb.edu (R.M.); 7 Department of Histopathology, Aarhus University Hospital, Palle Juul-Jensens Blvd. 99, 8200 Aarhus, Denmark

**Keywords:** fetal membranes, amniochorionic membranes, placenta, placental bed, preterm prelabor rupture of membranes, RNA sequencing, mRNA expression, immunohistochemistry, protein expression, biomarkers

## Abstract

Clinically, unique markers in fetal membrane cells may contribute to the search for biomarkers for preterm prelabor rupture of the fetal membranes (pPROM) in maternal blood. pPROM is associated with overwhelming inflammation and premature cellular senescence causing “biological microfractures” of the fetal membranes. We hypothesize that these pathological processes are associated with the shedding of fetal membrane cells into the maternal circulation. The aim of this study was to identify markers expressed exclusively in fetal membrane cells to facilitate their isolation, characterization, and determination of biomarker potential in maternal blood. We have (1), by their transcriptomic profile, identified markers that are upregulated in amnion and chorion tissue compared to maternal white blood cells, and (2), by immunohistochemistry, confirmed the localization of the differentially expressed proteins in fetal membranes, placenta, and the placental bed of the uterus. RNA sequencing revealed 31 transcripts in the amnion and 42 transcripts in the chorion that were upregulated. Among these, 22 proteins were evaluated by immunohistochemistry. All but two transcripts were expressed both on mRNA and protein level in at least one fetal membrane cell type. Among these remaining 20 proteins, 9 proteins were not significantly expressed in the villous and extravillous trophoblasts of the placenta.

## 1. Introduction

Globally, around 10% of deliveries are preterm (birth before 37 weeks of gestation) [1,2], which makes it the leading cause of mortality and morbidity among children below 5 years of age [3,4]. Preterm prelabor rupture of the fetal membranes (pPROM) causes about one-third of these cases [5]. Despite a vast amount of research in the field, currently there are no diagnostic tools to predict the risk of pPROM prior to its occurrence [6]. A commentary from the Biomarkers Group of PREBIC (Preterm Birth International Collaborative) has identified eleven sections of potential technologies for candidate markers in the prediction of spontaneous preterm birth, including pPROM [7]. Among these, cell-free RNA (cfRNA), proteomic analyses, and fetal membrane cells in maternal blood have been suggested as promising.

Most likely, pPROM is associated with a preterm mechanical weakening of the fetal membranes caused by abnormal inflammation or accelerated cellular senescence of the fetal membrane cells [8,9,10,11,12,13]. In normal term pregnancies, a similar, albeit not preterm senescence, is a physiologic requirement for natural progression of labor mechanisms [14,15]. Another characteristic of fetal membranes from pPROM deliveries is the increased number and size of the so-called “microfractures” in the amnion [16,17,18], which are characterized by (1) an altered morphology of the amnion epithelial cells, (2) a localized damage of the subepithelial basement membrane, (3) tunnels in the collagen rich extracellular matrix, and (4) the presence of migrating cells in the tunnels. Similar observations have been detected in the chorion, although it was more difficult to confirm because of its multilayer composition [16].

**Figure 1:**
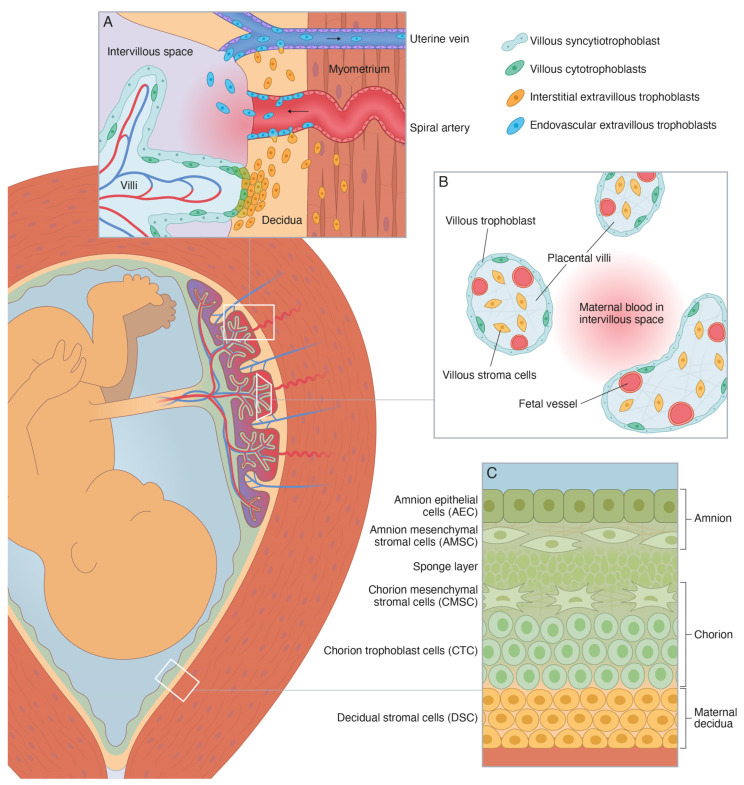
Schematic illustration of the perinatal tissues surrounding the fetus at term [20,21]. (**A**) Schematic illustration of the basal decidua and placental bed at term. The basal decidua and placental bed contain spiral arteries, uterine veins, and uterine lymphatics that are surrounded by interstitial extravillous trophoblasts (iEVT) and invaded by endovascular extravillous trophoblasts (vasEVT). (**B**) Schematic illustration of the peripheral villi at term. They are covered by a thin layer of fetus-derived villous trophoblasts (VT), primarily the syncytiotrophoblast, and contain a fetus-derived villous stroma of mesenchymal cells, fibroblasts, macrophages, and fetal capillaries and vessels with smooth muscle and endothelial cells. (**C**) Schematic illustration of the human amniochorionic membrane. The four fetus-derived layers comprise: (1) amnion epithelial cells (AEC), (2) amnion mesenchymal stromal cells (AMSC), (3) chorion mesenchymal stromal cells (CMSC), and (4) chorion trophoblast cells (CTC). The maternal layer contains (5) decidual stromal cells (DSC).

It has been suggested that the microfractures may constitute an important gateway for migration and transit of fetal membrane cells into the maternal circulation [19]. Such fetal membrane cells may originate from any of the four fetus-derived layers of the amniochorionic membrane (or chorion laeve), i.e., amnion epithelial cells, amnion mesenchymal cells, chorion mesenchymal cells, and chorion trophoblast cells (Figure 1) [20,21]. If so, they may be isolated from maternal blood using techniques equivalent to those used for isolation of other circulating fetal cells, such as hematopoietic and mesenchymal stem cells, lymphocytes, nucleated red blood cells, and extravillous trophoblasts (EVTs) [22,23,24].

Based on the above assumption of pPROM as “a disease of the fetal membranes” [19], we hypothesize that fetal membrane cells are present at increased concentrations in the maternal circulation prior to the event and thereby constitute predictive biomarkers for pPROM. To constitute a basis for the testing of this hypothesis, this study aimed to identify markers expressed by fetal membrane cells, but not by maternal blood cells or by placental cells. We have (1) by their transcriptomic profile, identified markers that are upregulated in amnion and chorion tissue compared to maternal white blood cells, and (2) by immunohistochemistry, confirmed the localization of the differentially expressed proteins in fetal membranes, placenta, and the placental bed of the uterus.

## 2. Results

### 2.1. Fetal Membrane mRNA Expression

Analysis of the RNA sequencing data using a customized pipeline based on the Tuxedo suite showed that, of the reported 90,000 transcripts, 2133 were upregulated in the amnion and 1904 were upregulated in the chorion as compared to maternal white blood cells. We chose all the genes having a Log2 fold change threshold of 9.4 for the amnion and 8.7 for the chorion, ultimately identifying 31 amnion markers and 42 chorion markers, of which 9 markers were upregulated both in amnion and chorion (Table 1). To refine the list of markers for further investigation, all upregulated transcripts underwent a literature review (Table S1), and based on this we selected 22 genes for immunohistochemistry (IHC) analysis (Table 2). Among these, 10 gene markers were upregulated in amnion, 10 gene markers were upregulated in chorion, and 2 gene markers were upregulated both in amnion and chorion. This rigorous selection process allowed us to focus on the most promising gene markers for our subsequent analysis.

### 2.2. Protein Localization

**Figure 2:**
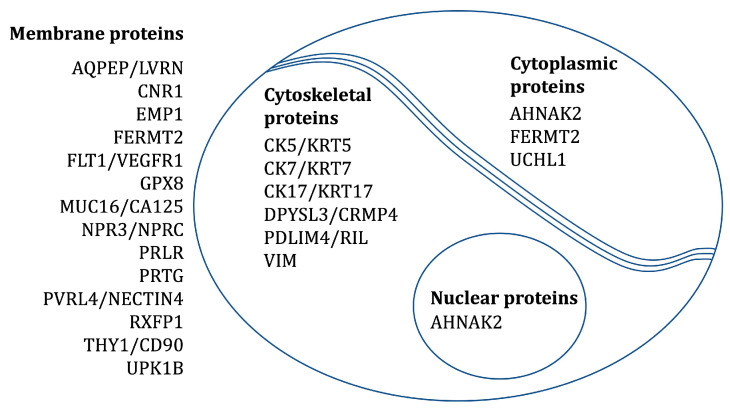
The expected subcellular locations of the investigated protein markers.

The localization of proteins corresponding to the abovementioned 22 transcripts was confirmed by IHC on tissues from fetal membranes, placenta, and the placental bed of the uterus. SHROOM3 and LPHN3 were excluded from the study because of failed antibody staining. CK7 and VIM were included as positive controls (CK7: epithelial cells and trophoblasts; VIM: mesenchymal stromal cells). Table 2 and Figure 2 provides an overview of the expected subcellular locations of the investigated protein markers.

The protein staining of selected cell populations was evaluated using the immunoreactivity score (IRS) (Table S2). This provides a score from 0 to 12 as a product of multiplication between (A) the proportion of positive cells (ranging from 0 to 4) and (B) the staining intensity (ranging from 0 to 3) of a specific cell population. In the present study, we defined a protein to be significantly expressed if the antibody stained over 50% of a given cell population (A ≥ 3) with at least a moderate reaction (B ≥ 2). A summary of the results is presented in Table 3.

**Figure 3:**
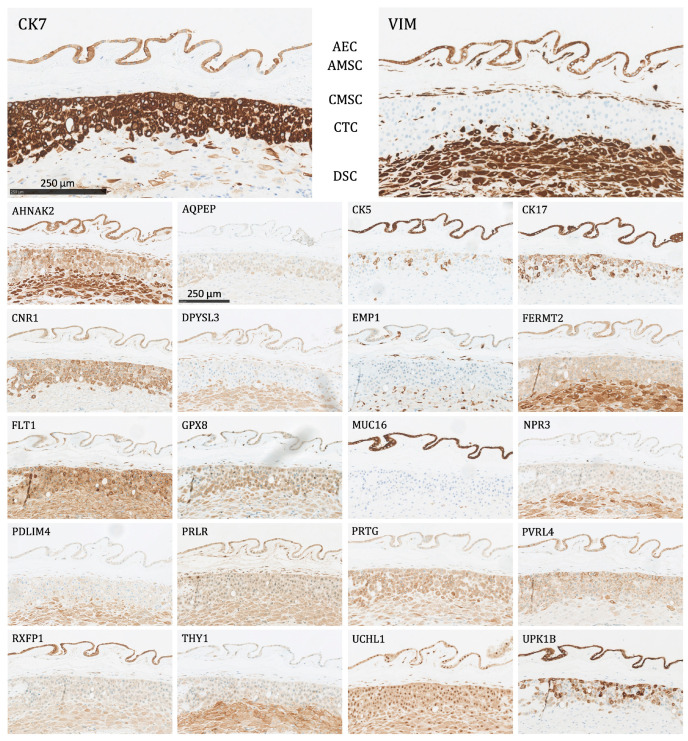
The protein staining of the fetal membrane cells for the 22 markers evaluated by immunohistochemistry. AEC: amnion epithelial cells, AMSC: amnion mesenchymal stromal cells, CMSC: chorion mesenchymal stromal cells, CTC: chorion trophoblast cells, DSC: decidual stromal cells. Proteins significantly expressed in AEC: AHNAK2, CK5, CK7, CK17, CNR1, DPYSL3, EMP1, FERMT2, FLT1, GPX8, MUC16, PRLR, PVRL4, RXFP1, UCHL1, UPK1B, and VIM; proteins significantly expressed in AMSC: AHNAK2, DPYSL3, EMP1, FLT1, GPX8, PRLR, RXFP1, UCHL1, and VIM; proteins significantly expressed in CMSC: AHNAK2, DPYSL3, EMP1, FLT1, GPX8, PDLIM4, PRLR, THY1, UCHL1, and VIM; proteins significantly expressed in CTC: CK7, CNR1, FLT1, GPX8, PRTG, PVRL4, UCHL1, and UPK1B; proteins significantly expressed in DSC: AHNAK2, DPYSL3, FERMT2, FLT1, GPX8, PDLIM4, PRTG, THY1, UCHL1, and VIM.

All 22 proteins except NPR3 and AQPEP were significantly expressed (as defined above) in at least one fetal membrane cell population (Figure 3). The following is a list of the different cell types and the proteins that were expressed in them:

- Amnion epithelial cells (AEC): Significant expression of AHNAK2, CK5, CK7, CK17, CNR1, DPYSL3, EMP1, FERMT2, FLT1, GPX8, MUC16, PRLR, PVRL4, RXFP1, UCHL1, UPK1B, and VIM.
- Amnion mesenchymal stromal cells (AMSC): Significant expression of AHNAK2, DPYSL3, EMP1, FLT1, GPX8, PRLR, RXFP1, UCHL1, and VIM.
- Chorion mesenchymal stromal cells (CMSC): Significant expression of AHNAK2, DPYSL3, EMP1, FLT1, GPX8, PDLIM4, PRLR, THY1, UCHL1, and VIM.
- Chorion trophoblast cells (CTC): Significant expression of CK7, CNR1, FLT1, GPX8, PRTG, PVRL4, UCHL1, and UPK1B.
- Decidual stromal cells (DSC): Significant expression of AHNAK2, DPYSL3, FERMT2, FLT1, GPX8, PDLIM4, PRTG, THY1, UCHL1, and VIM.

**Figure 4:**
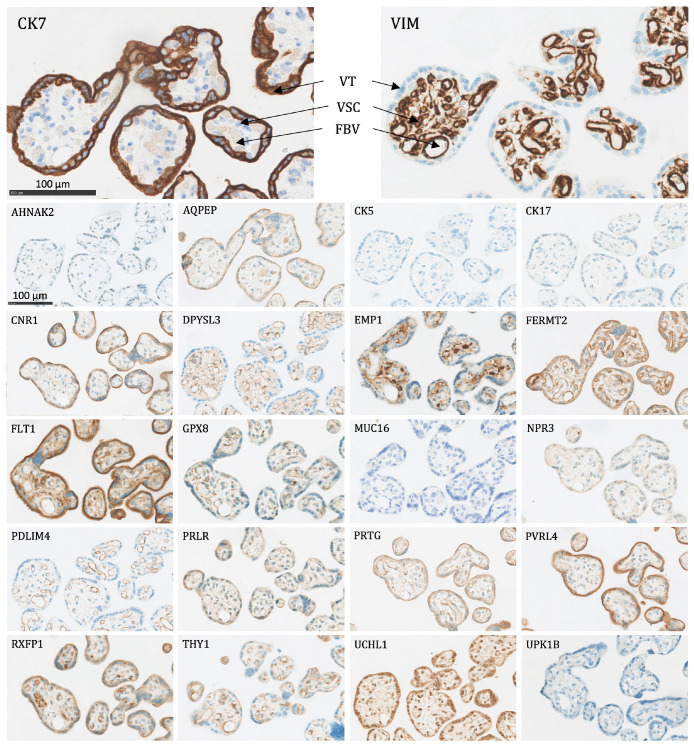
The protein staining of the peripheral villi of the placenta for the 22 markers evaluated by immunohistochemistry. VT: villous trophoblasts (primarily the syncytiotrophoblast), VSC: villous stromal cells, FBV: fetal blood vessel. Proteins significantly expressed in VT: CK7, CNR1, FERMT2, FLT1, PRTG, PVRL4, RXFP1, and UCHL1.

**Figure 5:**
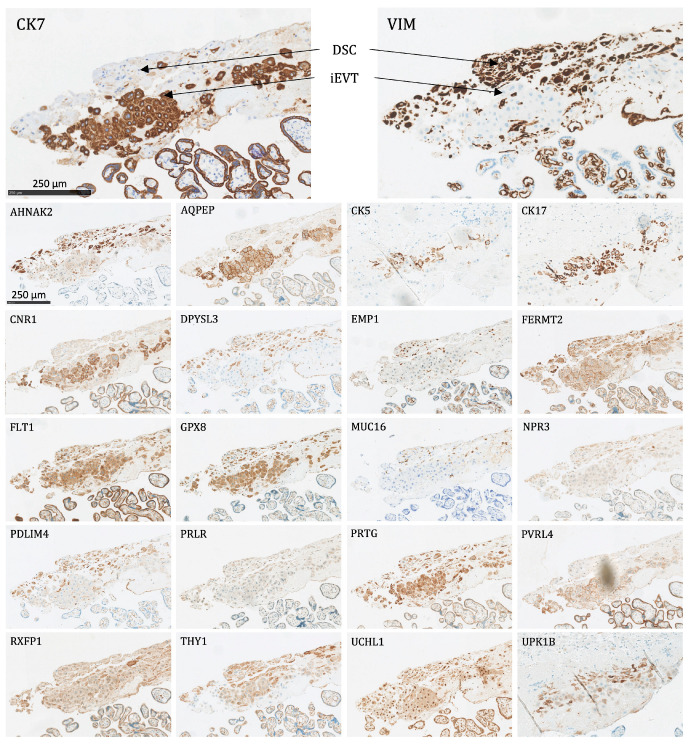
The protein staining of the interstitial extravillous trophoblasts of the basal plate and basal decidua for the 22 markers evaluated by immunohistochemistry. DSC: decidual stromal cells, iEVT: interstitial extravillous trophoblasts. Proteins significantly expressed in iEVT: AQPEP, CK7, CNR1, FERMT2, FLT1, GPX8, PRTG, PVRL4, THY1, and UCHL1.

**Figure 6:**
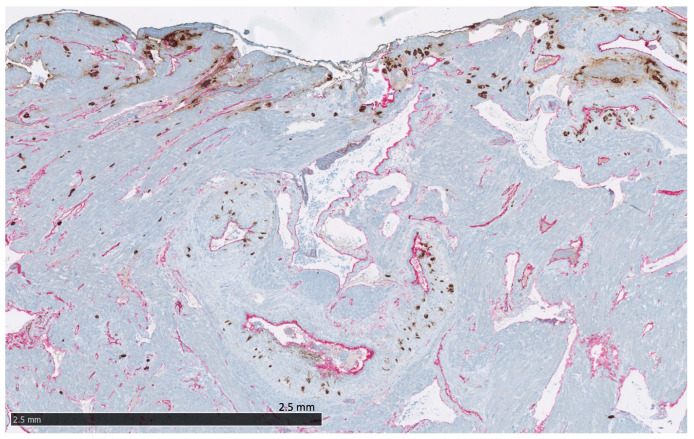
The placental bed of the uterus. CK7-positive interstitial and endovascular extravillous trophoblasts stained in brown invade the basal decidua and myometrium, from where they can reach the vessel wall of spiral arteries, veins, lymphatics, and glands. CD34-positive endothelium of vessels are stained in pink.

**Figure 7:**
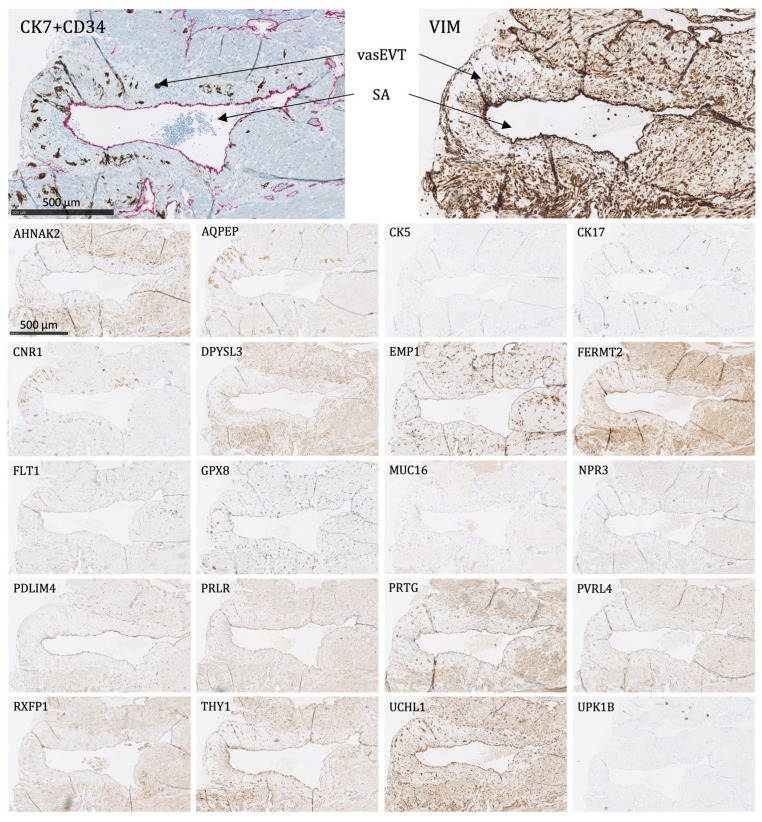
The protein staining of the endovascular extravillous trophoblasts of the placental bed for the 22 markers evaluated by immunohistochemistry. The endovascular extravillous trophoblasts are found in the tunica media of the vessel wall of the spiral arteries. For CK7, there is shown a double staining of CK7-positive endovascular extravillous trophoblasts in brown and CD34-positive endothelial cells in pink. SA: spiral artery, vasEVT: endovascular extravillous trophoblasts. Proteins significantly expressed in vasEVT: AQPEP, CK7, CNR1, FERMT2, FLT1, GPX8, PRTG, UCHL1, and UPK1B.

The following proteins were significantly expressed (as defined above) in the trophoblasts of the placenta and the placental bed of the uterus:

- Villous trophoblasts (VT), primarily the syncytiotrophoblast (Figure 4): Significant expression of CK7, CNR1, FERMT2, FLT1, PRTG, PVRL4, RXFP1, and UCHL1.
- Interstitial EVTs (iEVT) (Figure 5 and Figure 6): Significant expression of AQPEP, CK7, CNR1, FERMT2, FLT1, GPX8, PRTG, PVRL4, THY1, and UCHL1.
- Endovascular EVTs (vasEVT) (Figure 6 and Figure 7): Significant expression of AQPEP, CK7, CNR1, FERMT2, FLT1, GPX8, PRTG, UCHL1, and UPK1B.

Among the 20 significantly expressed proteins in fetal membrane cells, MUC16 was exclusively expressed in the fetal membranes (amnion epithelial cells) with a negative expression in all placental cell populations, i.e., the villous trophoblasts, the villous mesenchyme, and the EVTs (interstitial and endovascular). Furthermore, CK5 and CK17 were only mildly or moderately positive in the placental cell populations, and thus only significantly expressed in the fetal membranes. Though to some extent expressed in the villous mesenchyme, AHNAK2, DPYSL3, EMP1, PDLIM4, PRLR, and VIM were not significantly expressed in the villous trophoblasts and the EVTs.

## 3. Discussion

The objective of this study was to identify markers expressed by fetal membrane cells, but not by maternal blood cells or by placental cells. RNA sequencing revealed 31 transcripts in the amnion and 42 transcripts in the chorion that were upregulated when compared to maternal white blood cells. Among these, 22 proteins were evaluated by IHC. All but two proteins (AQPEP and NPR3) were significantly expressed in at least one fetal membrane cell type, and among these, nine were not significantly expressed in the villous and extravillous trophoblasts of the placenta. However, only MUC16 was expressed exclusively in the fetal membranes and more specifically in the amnion epithelium.

An important clinical perspective of our findings is the identification of biomarkers for the risk of pPROM. This is because the proteins assessed in this study may constitute a basis for the enrichment and isolation of possible fetal membrane cells that have entered the maternal blood through the microfractures in the fetal membranes, potentially arising several weeks before pPROM [19]. To address different approaches for the isolation of these cells, we included both cell surface markers (appropriate for magnetic activated cell sorting, MACS) and cytoplasmic markers (appropriate for fluorescence activated cell sorting, FACS) using commercially available antibodies (Table S1 and Table 2). However, when planning studies on this perspective, one must take into consideration that the results in the present study are from term pregnancies and might therefore not be fully converted to preterm pregnancies.

Even though no other research group has previously reported the isolation of circulating fetal membrane cells for the clinical prediction of pPROM, one study has isolated fetal mesenchymal stromal cells (that could originate from the fetal membranes) in 1 out of 20 maternal blood samples obtained immediately after termination of first trimester pregnancies [25]. Furthermore, some markers identified in the present study have previously been suggested for cell-free prenatal screening of pPROM. Thus, pPROM is associated with an increased FLT1 mRNA expression in both non-inflamed and inflamed fetal membrane tissues [26] and an abnormally high level of soluble FLT1 (sFLT-1) in early third-trimester maternal plasma [27]. The FLT1 protein (also known as VEGFR1), which plays an essential role in the development of embryonic vasculature and angiogenesis, has primarily been suggested as a biomarker for early-onset preeclampsia and fetal growth restriction [28,29,30]. In the present study, it was significantly expressed in all layers of the fetal membranes including decidua, as well as the villous trophoblast and the interstitial and endovascular EVTs. In addition, the RXFP1 mRNA expression was significantly increased in the chorion and decidua of fetal membranes from pPROM pregnancies compared to term pregnancies [31]. RXFP1 acts as a receptor for the hormone relaxin [32], which enhances collagenolytic activity, and hence an increase could lead to pPROM [33,34]. In the present study, the RXFP1 protein was significantly expressed in the two cell layers of the amnion (amnion epithelial cells and amnion mesenchymal stromal cells), although the RXFP1 mRNA expression was primarily found in the chorion, like in the above-mentioned study. To our knowledge, none of the remaining markers investigated in the present study have previously been used in the prenatal screening of pPROM.

MUC16 (also known as CA125) was the only marker exclusively expressed in the fetal membranes and more specifically in the amnion epithelium. It is a well-known tumor marker for epithelial ovarian malignancies [35]. However, it is also known to be expressed in the normal epithelia of the endometrium, benign ovarian cysts, and peritoneum [36]. This is why an increase can also be seen in cases of, e.g., pregnancy, endometriosis, and pelvic inflammatory disease [37,38]. As it can be increased in pregnancy, it has not previously been investigated as a biomarker for pPROM. However, due to its association with inflammatory reactions, one study found that amniotic fluid CA125 protein levels may serve as a predictor for intra-amniotic inflammation, microbial invasion of the amniotic cavity, and imminent delivery in cases of pPROM [39].

We conclude that the differentially expressed mRNAs and proteins identified in the present study are localized to the fetal membranes. These results may contribute to the uncovering of biomarkers in maternal blood for identification of pregnant women at risk of threatening rupture of membranes and pPROM. A compelling avenue for future research could involve comparing the findings with data obtained from tissues collected from individuals experiencing preterm premature rupture of membranes (pPROM). This comparative analysis could yield more comprehensive insights.

## 4. Materials and Methods

### 4.1. RNA Sequencing

Fetal membranes from one normal term (GA > 37) elective cesarean delivery, i.e., before the onset of labor, were collected from John Sealy Hospital at The University of Texas Medical Branch (UTMB) at Galveston, Texas, USA. They were mechanically separated into amnion and chorion, and chorion was cleaned off from decidua by cotton gauze. Biopsies of 6 mm from amnion and chorion were obtained, laid on dry ice, and sent to Aarhus University Hospital, Denmark. On arrival, the fresh frozen tissues were stored at −80 °C. The two biopsies and one buffy coat with maternal white blood cells from a normal late first trimester pregnancy were sent to BioXpedia A/S Denmark for RNA sequencing analysis. From each biopsy, 5 mg pieces were cut without thawing. RNA was extracted using the RNeasy UCP kit (Qiagen, Hilden, Germany) according to the manufacturer’s recommendations. RULT buffer of 350 µL was added along with two steel balls and homogenized in a TissueLyser (Qiagen). The homogenate was cleared by centrifugation and ethanol was added before binding the RNA to RNeasy spin columns. After several wash steps, RNA was eluted, quantified on a Nanodrop and checked for integrity on an Agilent Bioanalyzer. The RNA aliquots of selected amnion and chorion RNA samples as well as the white blood cells were shipped to Qiagen/Exiqon in Hilden, Germany, for paired-end sequencing using the Illumina TruSeq Stranded Total RNA Library Prep Kit (Illumina Inc., San Diego, CA, USA). Following sequencing, intensity correlation and base calling (into BCL files), FASTQ files were generated using bcl2fastq software, version 2.20.0 (Illumina Inc.) [40], which includes quality scoring of each individual base in a read. Data from all the samples showed a Q-score of greater than 30, indicating a high-quality read data. Thirty million reads were obtained from each sample and the genome mapping rate in amnion, chorion, and blood cells was 88.9%, 90.4%, and 83%, respectively. The number of identified genes per sample was calculated based on alignment to the reference genome.

The mRNA expression of amnion and chorion cells was compared to the mRNA expression of maternal white blood cells by binary logarithm (Table 1). All the highly expressed gene markers in amnion and chorion compared to maternal white blood cells underwent a literature review (Table S1). The subcellular location of the gene markers was evaluated using UniProt [41] and The Human Protein Atlas [42]. The markers located on the cell surface or the cell cytoplasm were further assessed with regards to their mRNA expression in cytotrophoblasts, syncytiotrophoblast, and extravillous trophoblast, and the protein expression (by IHC) of the trophoblastic cells and decidual cells using The Human Protein Atlas [42].

### 4.2. Immunohistochemistry

Fetal membranes and placental biopsies were obtained from five healthy women after normal vaginal delivery at term (GA > 37). Biopsies from the placental bed of the uterus were obtained from the uterotomy of three cesarean sections (GA 37–38) due to placenta previa covering the area for the uterotomy. All tissues were stored in 10% formalin and subjected to standard IHC procedures used for formalin-fixed, paraffin-embedded tissue.

Characteristics of the evaluated proteins and their corresponding antibodies used for IHC are shown in Table 2. A fully automated Ventana Benchmark Ultra Stainer Module (N750-BMKU-FS 05342716001) was used for IHC staining using the Ventana OptiView DAB IHC Detection Kit (Ventana Part Number 760–700, Roche GMMI 06396500001). The antibodies were optimized in terms of pretreatment, dilution, and incubation time as specified in Table 2.

All IHC stained slides were scanned with a VS200 slide scanner (Olympus, Center Valley, PA, USA) using a 20× objective and visualized using the software OlyVIA, version 3.3. (Olympus) [43] or scanned with a NanoZoomer 2.0-HT scanner (Hamamatsu Photonics, Shizuoka, Japan) and visualized using the software NDP.view2, version 2.8 (Hamamatsu) [44]. The protein staining was manually evaluated using the immunoreactivity score (IRS) [45,46,47,48]. It quantifies (A) the percentage of positive cells (0: no positive cells, 1: < 10% positive cells, 2: 10–50% positive cells, 3: 51–80% positive cells, and 4: > 80% positive cells) and (B) the staining intensity (0: no color reaction, 1: mild reaction, 2: moderate reaction, and 3: intense reaction). By multiplying these two variables, it provides a score from 0 to 12, where the staining is defined as IRS 0–1: negative, IRS 2–3: mildly positive, IRS 4–8: moderately positive, and IRS 9-12: strongly positive. A protein was defined to be significantly expressed if the corresponding antibody stained more than 50% of a given cell population (A ≥ 3) with at least a moderate reaction (B ≥ 2). The staining was initially evaluated by E.M. and afterwards reviewed by B.H., who defined the final scores.

## 5. Patents

The authors R.M., R.S., K.R. and L.H. have filed a patent application on using the markers from the present study for isolation and identification of fetal membrane cells in maternal blood.

## Figures and Tables

**Table 1:** Upregulated gene markers in amnion and chorion compared to maternal white blood cells by binary logarithm. The markers with the highest expression are listed on top.

Amnion Markers	Log2 Fold Change	Chorion Markers	Log2 Fold Change
*IGF2*	19.5	*IGF2*	15.6
*MUC16*	13.4	*THY1*	14.1
*CK5*	11.7	*DCN*	12.8
*TSPAN1*	11.6	*DIO2*	11.9
*IGFBP3*	11.3	*IGFBP3*	11.8
*SERPINB10*	11.3	*IGFBP2*	11.6
*UPK1B*	11.3	*SPARCL1*	11.1
*CADPS2*	11.2	*NNMT*	10.9
*LAMC2*	11.2	*LPHN3*	10.9
*AHNAK2*	11.1	*CRYAB*	10.9
*EMP1*	11.0	*PEG3*	10.3
*FN1*	10.8	*FLT1*	10.2
*FBN1*	10.7	*GPX8*	9.9
*MET*	10.7	*FBN1*	9.8
*PVRL4*	10.6	*NPR3*	9.8
*A2ML1*	10.4	*AOC1*	9.8
*DSP*	10.3	*ITGB8*	9.7
*THSD4*	10.2	*RXFP1*	9.5
*CRYAB*	10.2	*SPOCK1*	9.5
*CK17*	10.1	*CYP11A1*	9.5
*CK18*	10.1	*COL4A2*	9.4
*PDLIM4*	10.1	*CK18*	9.4
*COL17A1*	10.0	*CNR1*	9.4
*PRTG*	9.8	*SEMA3A*	9.4
*DKK3*	9.8	*SERPINE1*	9.4
*PLS3*	9.8	*IL1R1*	9.4
*COL1A2*	9.7	*FBLN1*	9.3
*GPX8*	9.6	*COL1A2*	9.3
*DPYSL3*	9.5	*UCHL1*	9.3
*TPPP3*	9.5	*RAI2*	9.2
*SHROOM3*	9.4	*TGM2*	9.2
		*PRLR*	9.2
		*FSTL3*	9.1
		*SERPINB10*	9.1
		*BCAR1*	9.1
		*THSD4*	9.0
		*PRTG*	9.0
		*FERMT2*	8.9
		*PKP2*	8.9
		*P4HA2*	8.8
		*TEAD1*	8.8
		*AQPEP*	8.8

**Table 2:** Characteristics of the selected proteins and their corresponding antibodies (including pretreatment and dilution) used for immunohistochemistry. The 22 upregulated genes were selected based on their mRNA expression in the fetal membranes (amnion or chorion) and their subcellular location (cell surface or cytoplasm). CK7 and VIM were included as positive cell controls (CK7: epithelial cells and trophoblasts; VIM: mesenchymal stromal cells). CD34 was used to confirm the presence of endothelial cells and hence vessels in the placental bed of the uterus.

Protein	Fetal Membrane	Subcellular Location	Antibody ^1^	Pretreatment ^2^	Dilution ^3^
AHNAK2 (Protein AHNAK2)	Amnion	Cytoplasm	Abcam, ab224061 (rabbit, polyclonal)	CC2 32 min	1:500
AQPEP/LVRN (Aminopeptidase Q)	Chorion	Surface	Abcam, ab185345 (rabbit, polyclonal)	CC1 32 min	1:500
CD34 (Hematopoietic progenitor cell antigen CD34)	-	-	Ventana-Roche, 790-2927 (mouse, monoclonal QBEnd/10)	CC1 40 min	RTU
CNR1 (Cannabinoid receptor 1)	Chorion	Surface	Abcam, ab23703 (rabbit, polyclonal)	CC2 32 min	1:200
CK5/KRT5 (Keratin, type II cytoskeletal 5)	Amnion	Cytoplasm	Novocastra-Leica, NCL-L-CK5 (mouse, monoclonal XM26)	CC1 32 min	1:100
CK7/KRT7 (Keratin, type II cytoskeletal 7)	-	Cytoplasm	Ventana-Roche, 790-4462 (rabbit, monoclonal SP52)	CC1 40 min	RTU
CK17/KRT17 (Keratin, type I cytoskeletal 17)	Amnion	Cytoplasm	Ventana-Roche, 790-4560 (rabbit, monoclonal SP95)	CC1 32 min	RTU
DPYSL3/CRMP4 (Dihydropyrimidinase-related protein 3)	Amnion	Cytoplasm	Abcam, ab244319 (rabbit, polyclonal)	CC2 32 min	1:1000
EMP1 (Epithelial membrane protein 1)	Amnion	Surface	LSBio, LS-B12859 (rabbit, polyclonal)	CC1 32 min	1:500
FERMT2 (Fermitin family homolog 2)	Chorion	Surface	Abcam, ab254535 (mouse, monoclonal 3A3.5)	CC1 32 min	1:500
FLT1/VEGFR1 (Fms-like tyrosine kinase 1/Vascular endothelial growth factor receptor 1)	Chorion	Surface	Abcam, ab32152 (rabbit, monoclonal Y103)	CC2 32 min	1:50
GPX8 (Probable glutathione peroxidase 8)	Amnion Chorion	Surface	Abcam, ab183664 (rabbit, polyclonal)	CC1 32 min	1:50
LPHN3/ADGRL3 (Adhesion G protein-coupled receptor L3)	Chorion	Surface	Abcam, ab140843 (rabbit, polyclonal)	Failed to be stained	
MUC16/CA125 (Mucin-16)	Amnion	Surface	Ventana-Roche, 760-2610 (mouse, monoclonal OC125)	CC1 48 min	RTU
NPR3/NPRC (Atrial natriuretic peptide receptor 3)	Chorion	Surface	Abcam, ab97389 (rabbit, polyclonal)	CC1 32 min	1:250
PDLIM4/RIL (PDZ and LIM domain protein 4)	Amnion	Cytoplasm	Abcam, ab251701 (rabbit, polyclonal)	CC2 32 min	1:20
PRLR (Prolactin receptor)	Chorion	Surface	Abcam, ab2773 (mouse, monoclonal T6)	CC1 16 min	1:500
PRTG (Protogenin)	Amnion Chorion	Surface	LSBio, LS-C817053 (rabbit, polyclonal)	CC1 32 min	1:100
PVRL4 (Nectin-4)	Amnion	Surface	Abcam, ab155692 (rabbit, polyclonal)	CC1 32 min	1:100
RXFP1 (Relaxin receptor 1)	Chorion	Surface	Sigma-Aldrich, HPA027067 (rabbit, polyclonal)	CC1 32 min	1:100
SHROOM3 (Protein Shroom 3)	Amnion	Surface	Abcam, ab151009 (rabbit, polyclonal)	Failed to be stained	
THY1/CD90 (Thy-1 membrane glycoprotein)	Chorion	Surface	Abcam, ab133350 (rabbit, monoclonal EPR3133)	CC1 32 min	1:250
UCHL1 (Ubiquitin carboxyl-terminal hydrolase isozyme L1)	Chorion	Cytoplasm	Dako Agilent, Z0458 (rabbit, polyclonal)	CC1 40 min	1:200
UPK1B (Uroplakin-1b)	Amnion	Surface	Abcam, ab263454 (mouse, monoclonal UPK1B/3081)	CC2 32 min	1:250
VIM (Vimentin)	-	Cytoplasm	Ventana-Roche 790-2917(mouse, monoclonal V9)	CC1 24 min	RTU

^1^ DAP was used as chromogen for all antibodies except for CD34, where Fast Red was used as chromogen. ^2^ CC1: ULTRA Cell Conditioning Solution, Ventana 950-224/05424569001. CC2: ULTRA Cell Conditioning Solution, Ventana 950-223/05424542001. ^3^ RTU: Ready-To-Use antibody.

**Table 3:** The protein staining of selected fetus-derived cell populations. The 22 markers were evaluated on five fetal membrane biopsies where amnion was detached and missing in two of them, five placental biopsies, and three biopsies from the placental bed of the uterus.

Protein	AEC	AMSC	CMSC	CTC	VT	iEVT	vasEVT
	*N* = 3	*N* = 3	*N* = 5	*N* = 5	*N* = 5	*N* = 3	*N* = 3
AHNAK2	**12.0**	**12.0**	**12.0**	4.4	0.0	4.7	1.0
AQPEP	0.0	2.3	1.2	4.0	7.2	**11.0**	**12.0**
CK5	**12.0**	0.0	0.0	3.4	0.0	2.0	0.0
CK7	**10.0**	0.0	0.0	**12.0**	**12.0**	**12.0**	**12.0**
CK17	**12.0**	0.0	0.0	6.8	0.0	4.0	3.0
CNR1	**8.0**	4.0	5.8	**9.0**	**12.0**	**12.0**	**12.0**
DPYSL3	**7.3**	**12.0**	**12.0**	0.8	0.0	0.7	1.0
EMP1	**9.3**	**10.0**	**10.2**	2.4	4.4	0.0	0.0
FERMT2	**8.0**	5.3	6.2	6.4	**10.4**	**6.7**	**12.0**
FLT1	**8.0**	**12.0**	**11.2**	**11.4**	**12.0**	**11.0**	**8.0**
GPX8	**10.7**	**7.0**	**8.4**	**10.6**	0.6	**9.0**	**10.7**
MUC16	**12.0**	0.0	0.0	1.4	0.0	0.0	0.0
NPR3	1.7	3.0	3.0	2.2	2.6	1.3	1.7
PDLIM4	1.3	4.3	**7.6**	3.0	0.0	2.0	1.0
PRLR	**7.0**	**11.0**	**11.4**	3.0	3.4	4.0	4.7
PRTG	5.3	2.3	4.6	**10.8**	**11.2**	**11.0**	**12.0**
PVRL4	**12.0**	1.0	1.4	**9.6**	**12.0**	**7.7**	4.0
RXFP1	**12.0**	**6.0**	5.4	4.0	**10.4**	5.0	5.3
THY1	2.3	6.3	**8.0**	3.0	1.0	7.3	5.3
UCHL1	**12.0**	**12.0**	**12.0**	**12.0**	**12.0**	**12.0**	**12.0**
UPK1B	**12.0**	0.0	0.0	**9.0**	0.0	7.0	**9.0**
VIM	**11.0**	**12.0**	**12.0**	0.0	0.0	0.0	0.0

Marked gray box with bold numbers: More than 50% of a given cell population was stained with at least a moderate reaction (i.e., significantly expressed proteins). Avg. IRS 0–1: negative, avg. IRS 2–3: mildly positive, avg. IRS 4–8: moderately positive, avg. IRS 9–12: strongly positive. AEC: amnion epithelial cells, AMSC: amnion mesenchymal stromal cells, CMSC: chorion mesenchymal stromal cells, CTC: chorion trophoblast cells, VT: villous trophoblast, iEVT: interstitial extravillous trophoblasts, vasEVT: endovascular extravillous trophoblasts.

## Data Availability

All other data from this study not presented within the article or supplementary materials are available to other researchers upon written request to the corresponding author.

## Supplementary Materials

The following supporting information can be downloaded at: https://www.mdpi.com/article/10.3390/ijms242115826/s1.
